# Contextual MEG and EEG Source Estimates Using Spatiotemporal LSTM Networks

**DOI:** 10.3389/fnins.2021.552666

**Published:** 2021-03-09

**Authors:** Christoph Dinh, John G. Samuelsson, Alexander Hunold, Matti S. Hämäläinen, Sheraz Khan

**Affiliations:** ^1^Athinoula A. Martinos Center for Biomedical Imaging, Massachusetts General Hospital, Charlestown, MA, United States; ^2^Department of Radiology, Massachusetts General Hospital (MGH), Charlestown, MA, United States; ^3^Institute for Medical Engineering, Research Campus STIMULATE, Otto-von-Guericke University, Magdeburg, Germany; ^4^Harvard Medical School, Boston, MA, United States; ^5^Harvard-MIT Division of Health Sciences and Technology (HST), Massachusetts Institute of Technology, Cambridge, MA, United States; ^6^Institute of Biomedical Engineering and Informatics, Technische Universität Ilmenau, Ilmenau, Germany

**Keywords:** MEG, EEG, source estimation, spatiotemporal source estimation, spatial filtering, grid-based Markov localization, LSTM, deep learning

## Abstract

Most magneto- and electroencephalography (M/EEG) based source estimation techniques derive their estimates sample wise, independently across time. However, neuronal assemblies are intricately interconnected, constraining the temporal evolution of neural activity that is detected by MEG and EEG; the observed neural currents must thus be highly context dependent. Here, we use a network of Long Short-Term Memory (LSTM) cells where the input is a sequence of past source estimates and the output is a prediction of the following estimate. This prediction is then used to correct the estimate. In this study, we applied this technique on noise-normalized minimum norm estimates (MNE). Because the correction is found by using past activity (context), we call this implementation Contextual MNE (CMNE), although this technique can be used in conjunction with any source estimation method. We test CMNE on simulated epileptiform activity and recorded auditory steady state response (ASSR) data, showing that the CMNE estimates exhibit a higher degree of spatial fidelity than the unfiltered estimates in the tested cases.

## Introduction

Magneto- and electroencephalography (M/EEG) have excellent sub-millisecond temporal resolution but limited spatial resolution. The most commonly used M/EEG distributed source estimation methods, e.g., MNE, dSPM, and sLORETA, are linear and source estimates are derived time-sample by time-sample, without considering the temporal sequence (Hamalainen and Ilmoniemi, 1994; Dale et al., 2000; Pascual-Marqui, 2002). In other words, these methods fit their source estimates directly to the sensor data without assuming any relationship between the neuronal current distributions across time. This inverse problem is ill-posed because different current distributions can produce the same or similar electric potentials and magnetic fields around the head as detected by the limited amount of M/EEG sensors (Helmholtz, 1853; Hämäläinen et al., 1993). The ill-posedness of the inverse problem along with the low SNR in M/EEG recordings cause the limited spatial resolution of the MEG and EEG technologies (Samuelsson et al., 2020).

A few different approaches have been developed to deal with this ill-posedness. The most common approach involves penalty terms on the source amplitude (regularization) or constraints limiting the solution space of the inverse problem. Constraining the solution space can be achieved by, e.g., assuming spatial smoothness (Pascual-Marqui et al., 1994; Dinh et al., 2015, 2018) or imposing focal estimates (Gorodnitsky et al., 1995). Bayesian methods that employ an estimated source covariance matrix as a prior have also been employed to restrict the solution space to sparse source reconstructions (Phillips et al., 2005; Mattout et al., 2006) or by computing posterior distributions based on hierarchical priors (Sato et al., 2004; Nummenmaa et al., 2007; Costa et al., 2017). These methods, however, commonly assume that the prior probability distribution of the sources is independent of time. This assumption ignores the temporal structure of the underlying neural activity that could be used to help reduce the ill-posedness of the inverse problem by constraining the solution space.

It is well-known that the human brain is intricately interconnected and several studies have shown that dynamic spatiotemporal interactions are central features of brain activity (Khan et al., 2015). For example, intracranial cortical recordings show strong local spatial correlations within 10 mm along the cortex (Bullock et al., 1995; Destexhe et al., 1999; Leopold et al., 2003) and physiologically motivated spatiotemporal models of neuronal networks have had success in explaining EEG and MEG data (Gross et al., 2001; Jirsa et al., 2002; Wright et al., 2003; Izhikevich and Edelman, 2008). These observations indicate that neural activity has a distinct spatiotemporal dynamics meaning that the brain state at any given time is a function of past brain activity, i.e., its context (Kozhemiako et al., 2020).

There have been efforts to include spatiotemporal dynamics in M/EEG source estimation but these methods have assumed certain constrained spatiotemporal interactions. One such example is the use of Bayesian source estimation methods that incorporate temporal smoothness constraints, which specify various prior distributions for the sources in space and time (Baillet and Garnero, 1997; Greensite, 2003; Somersalo et al., 2003; Friston et al., 2008; Limpiti et al., 2008; Trujillo-Barreto et al., 2008; Zumer et al., 2008; Bolstad et al., 2009; Ou et al., 2009; Sorrentino et al., 2009; Lucka et al., 2012; Vivaldi and Sorrentino, 2016; Calvetti et al., 2019). Mixed-Norm estimates have been introduced that impose spatial stationarity of the source estimates within a given time window and quasinorm penalties to promote spatial sparsity (Gramfort et al., 2012; Gramfort et al., 2013b; Strohmeier et al., 2014). Linear state-space models have also been employed that either apply temporally independent approximations (Galka et al., 2004) or a parametric approach (Long et al., 2011) to reduce their computational burden. Recent studies have introduced more realistic spatiotemporal dynamic models using Kalman filters, which take local cortical interactions into account (Lamus et al., 2012; Pirondini et al., 2018) by assuming a linear relationship between subsequent samples. This approach implicitly includes the estimation history but with the limiting assumption that past activity is linearly related to the subsequent activation and directly manifests in the source estimate. Although these models have shown promise, the potential of incorporating non-linear long range dynamic interactions without strict *a priori* assumptions to improve inverse solutions has remained largely unexplored.

Meanwhile, recent advances in machine learning have focused on sequential data sets, e.g., recurrent neural networks (RNN), enabling contextual data recognition (LeCun et al., 2015; Schmidhuber, 2015). These new contextual capabilities have been demonstrated to significantly improve classification accuracy in natural language processing (NLP) and have been successfully applied in, e.g., text and speech recognition (Graves et al., 2013; Chorowski et al., 2015). Although some studies have used machine learning techniques to classify various brain states or seizures based on MEG and particularly EEG data, most studies have done so in sensor space and the use of machine learning techniques in the M/EEG inverse problem has yet to be fully explored (Hofmann et al., 2018; Ali et al., 2019; Yu et al., 2019).

Here we investigate whether contextual machine learning techniques can be applied to reduce the ill-posedness of the M/EEG inverse problem, thus utilizing the superior temporal resolution of M/EEG to increase the spatial fidelity of source estimates. This approach thus constitutes a spatiotemporal inverse method that is based on deep learning without too strong explicit *a priori* modeling assumptions, except for those intrinsic to MNE, which has been the main focus of previous spatiotemporal inverse methods. In our approach, source estimates are spatially filtered, or “corrected,” by a prediction that has been generated by a network of long short-term memory (LSTM) cells (Hochreiter and Schmidhuber, 1997) from a sequence of previous source estimates. LSTM cells constitute a special type of RNN which has consistently shown success when applied to data with a temporal structure, e.g., in natural language processing and grid-based markov localization problems, and is thus a suitable candidate for a spatiotemporal inverse operator.

In this study we implemented the technique together with noise-normalized minimum norm estimates (MNE), and therefore call this implementation Contextual Minimum Norm Estimates (CMNE). Importantly, this method can also be described in the framework of linear algebra, where the weighing vector is being updated in a time-dependent manner using the prior context. Conceptually, the main advantage of the CMNE approach over recursive Bayesian filters like the Kalman filter is that its model is not explicitly defined and can instead be learned from the data; RNNs are thus more general than Kalman filter approaches. We tested our CMNE implementation on simulated epileptiform and recorded M/EEG data from auditory steady state response (ASSR) experiments. An earlier preprint version of this work was posted at arXiv (Dinh et al., 2019).

## Materials and Methods

### Contextual Estimates

We begin by describing our method and the specific implementation used in this study. The implementation of the presented method can be found at https://github.com/chdinh/cmne. We then describe the data analysis and training of the LSTM network. We denote vectors and scalars by lowercase and matrices by uppercase characters.

M/EEG signals are linked to their neural sources by a time-invariant gain matrix *G*, which incorporates the forward model:

(1) $$y_{t}=Gx_{t}+n_{t},$$

where the vector *y*_t_ represents the sensor data at time *t*, the vector *x*_t_ represents the true source distribution, and *n*_t_ is the noise. In the following, we will be using the whitened measured signals ${\tilde{y}}_{\text{t}}$ and whitened gain matrix $\tilde{\text{G}}$,

(2) $${\tilde{y}}_{t}=C_{n}^{-1/2}y_{t}$$

and

(3) $$\tilde{G}=C_{n}^{-1/2}G,$$

where *C*_n_ is the noise covariance matrix. Most source estimates ${\hat{x}}_{\text{t}}$ are found by minimizing a cost function,

(4) $${\hat{x}}_{t}=\arg\min_{x_{\text{t}}}\left({||{\tilde{y}}_{t}-\tilde{G}x_{t}||}_{2}^{2}+f\left(x_{t}\right)\right)$$

where the first term is the Euclidean norm of the difference between the measured data ${\tilde{y}}_{\text{t}}$ and the predicted signal $\tilde{G}x_{\text{t}}$ based on the model $\tilde{G}$ and source distribution *x*_t_, while *f*(*x*_t_) incorporates *a priori* assumptions or regularization. In MNE, $f\left(x_{\text{t}}\right)=\lambda^{2}x_{\text{t}}^{T}C_{\text{R}}^{-1}x_{\text{t}}$, where *C*_R_ is the source covariance matrix and λ^2^ is the Tikhonov regularization parameter, which was set to λ^2^ = 1/SNR^2^ = 1/9, assuming an SNR of 3. The solution to this minimization problem can be written as a product of the measured data ${\tilde{y}}_{\text{t}}$ and an inverse kernel *K*,

(5) $${\hat{x}}_{t}=K\tilde{y_{t}},$$

(6) $$K=C_{R}{\tilde{G}}^{T}{\left(\tilde{G}C_{R}{\tilde{G}}^{T}+\lambda^{2}I\right)}^{-1},$$

which is thus time-invariant and is applied sample-wise to compute the source estimate ${\hat{x}}_{\text{t}}$ from each measurement ${\tilde{y}}_{\text{t}}$. In dynamic statistical parametric mapping (dSPM) (Dale et al., 2000), the inverse kernel *K* is normalized with respect to the noise energy mapped to each source;

(7) $$K_{\text{dSPM}}=W_{\text{dSPM}}K,$$

where *W*_*dSPM*_ is a diagonal matrix;

(8) $$W_{{\text{dSPM}}_{i,i}}=\frac{1}{\sqrt{{\text{diag}}_{i}\left(KC_{n}K^{T}\right)}}.$$

In this study, the dSPM estimates were rectified and then normalized by z-scoring;

(9) $${\hat{q}}_{t_{i}}=\left(\left|{\hat{x}}_{t_{i}}\right|-\mu \left(\left|{\hat{x}}_{t_{i}}\right|\right)\right)/\sigma \left(\left|{\hat{x}}_{t_{i}}\right|\right).$$

In CMNE, the estimates are then reweighted with a time-dependent diagonal weighting matrix whose diagonal elements are the output of the LSTM network;

(10) $$b_{t}=W_{t}^{CMNE}{\hat{q}}_{t},$$

(11) $$\begin{array}{c} \text{diag}\left(W_{t}^{CMNE}\right)=\left|\text{LSTM}\left({\left\{b_{i}\right\}}_{i=t-k,\ldots ,t-1}\right)\right|\\ /{\max\left(\left|\text{LSTM}\left({\left\{b_{i}\right\}}_{i=t-k,\ldots ,t-1}\right)\right|\right)|}_{t\geq k}, \end{array}$$

(12) $$W_{t}^{CMNE}={I|}_{t=0..k-1}$$

where *b*_t_ is the final contextual estimate, LSTM is our network of LSTM cells, *k* is the number of LSTM cells in our network, which is a hyperparameter, and $W_{\text{t}}^{CMNE}$ is a diagonal matrix whose diagonal entries is the prediction output of the LSTM network after the first *k* time steps, when the LSTM network has sufficient history to make a prediction, and the identity matrix before then. After the first *k* timesteps, the CMNE estimate itself is used as input to the LSTM network, forming a Markov chain;

(13) $$b_{t}=W_{t}^{CMNE}\left(b_{i=t-1,\ldots ,t-k}\right){\hat{q}}_{t}.$$

**FIGURE 1:**
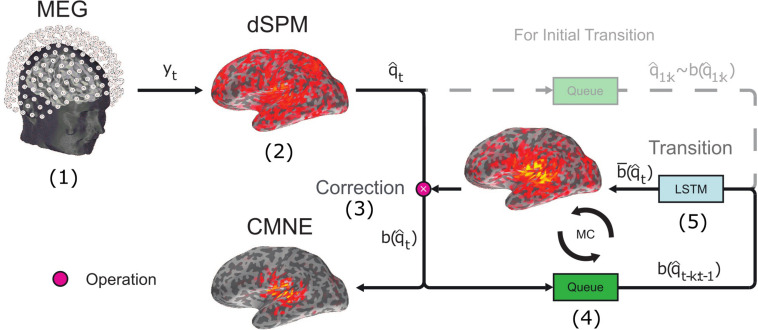
Schematic illustration of CMNE outlining the major steps. (1) M/EEG data are measured, saved and processed as per standard protocol. (2) Based on the measurement data *y*_*t*_, a source estimate ${\hat{q}}_{t}$ is found by dSPM. (3) The dSPM source estimate ${\hat{q}}_{t}$ is corrected by a prediction $\bar{b}\left({\hat{q}}_{t}\right)$ of what the estimate is expected to be based upon previous activations. This prediction $\bar{b}\left({\hat{q}}_{t}\right)$ is found by an LSTM network (explained in step 5). The correction is an elementwise product ∗ of the source estimate ${\hat{q}}_{t}$ and the prediction $\bar{b}\left({\hat{q}}_{t}\right)$, and the resulting product $b\left({\hat{q}}_{t}\right)$ is the CMNE estimate: $b\left({\hat{q}}_{t}\right)={\hat{q}}_{t}*\bar{b}\left({\hat{q}}_{t}\right)$. (4) The CMNE estimate $b\left({\hat{q}}_{t}\right)$ is put into a stack together with the k previous activations $b\left({\hat{q}}_{t-k:t}\right)$. (5) This stack of previous CMNE estimates is given to the LSTM network to predict the next activation $\bar{b}\left({\hat{q}}_{t+1}\right)$. This forms an iterative circle, i.e., a Markov chain (MC) (Dinh, 2015). In the first *k* time steps there is not enough prior history to make a prediction and the dSPM estimates are therefore not corrected and used as input to the LSTM network instead of the CMNE estimates.

In this approach, we thus use two spatial filters, first the filter found by the inverse of the noise covariance matrix (dSPM) and secondly the spatial filter given by the LSTM prediction which contains the contextual information. These two filters serve different functionalities; dSPM gives statistical scores with respect to the baseline while keeping the weights static. In contrast, the CMNE weights are updated in a recursive fashion. This algorithm is illustrated in Figure 1.

**FIGURE 2:**
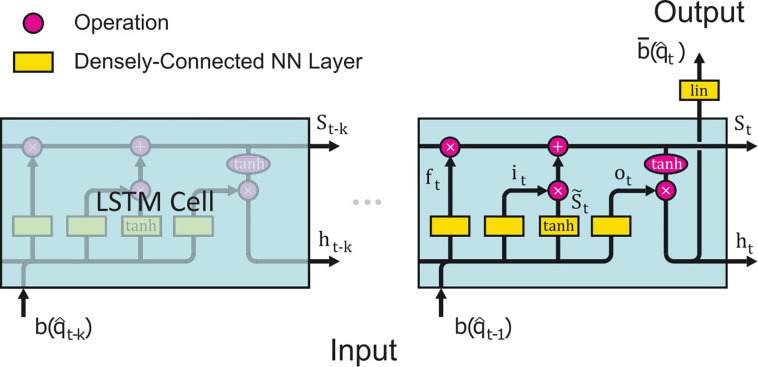
Schematic illustration of the LSTM network architecture. The basic structure encompasses *k* LSTM cells connected in series, where the cell *i* receives input from the previous cell in the form of the cell state *S*_*i–1*_ (Eq. 18) and hidden cell state *h*_*i–1*_ (Eq. 19), and then ends with a fully connected NN layer, which outputs the prediction of the LSTM network $\bar{b}.$ Apart from the input from the previous cell, each LSTM cell also receives input from the stack of previous estimates *b*_*i*_. Each LSTM cell consists of four fully connected NN layers (yellow boxes) which all have the same complexity *d* = 1,280: (i) *f*_*t*_ (Eq. 14), the “forget gate layer,” is an NN with a sigmoid activation function. This layer gates the previous cell states 0≤*S*_*t*−1_≤1 (Eq. 18) depending on *h*_*t–1*_ (Eq. 19) and the previous estimate $b\left({\hat{q}}_{t-1}\right)$ (Eq. 20). (ii) *i*_*t*_ (Eq. 15), the “input gate layer,” is also a sigmoid layer with the same inputs as *f*_*t*_. It determines which cell state values to update. (iii) ${\tilde{S}}_{t}$ (Eq. 16) is the subsequent fully connected NN layer with a *tanh* activation function, which creates candidate cell state values gated by *i*_*t*_. The state *S*_*t*_ of the current cell is formed by “forgetting” outdated information of the previous cell state *S*_*t–1*_ through a multiplication with *f*_*t*_ followed by an update with the gated new cell state candidates $i_{t}*{\tilde{S}}_{t}$. (iv) *o*_*t*_ is a fully connected NN layer activated by a sigmoid function. This layer decides which cell states to output. It gates the *tanh* scaled cell state *S*_*t*_. Because all NN layers have the same number of neurons *d* except for the final output layer, *S*_*t*_ and *h*_*t*_ are both *d*-dimensional vectors, see section “Contextual Estimates” for more details. The illustration is adapted from Olah (2015).

The employed LSTM network consists of an LSTM cell sequence, shown in Figure 2, where each cell *i* has a source estimate of the corresponding past time step $b\left({\hat{q}}_{t-i}\right)$ as its input, which was standardized by z-scoring. For the first *k* time steps, before we have enough previous estimates to generate a prediction, the non-contextual dSPM estimates ${\hat{q}}_{\text{i}}$ are used (Figure 1). Each LSTM cell consists of four fully connected neural networks (NN); the “forget gate layer,” “input gate layer,” “candidate cell state layer,” and “output gate layer.” All of those networks have sigmoidal activation functions except for the candidate cell state layer which has tanh activation functions. The actual output *h*_t_ of the LSTM cell is a filtered subset of the cell state *S*_t_. The number of neurons in each neural network layer *d* is another hyperparameter, in addition to the number of LSTM cells *k*. There are thus two hyperparameters in this network; *k* and *d*. The LSTM network is followed by a densely connected neural network layer with linear activation functions, i.e., a linear transformation with adaptable entries, mapping the output of the last LSTM cell *h*_*t*_ to the prediction $\bar{b_{t}}$ (Figure 2). The processing steps of each LSTM cell can thus be summarized in the following equations (Hochreiter and Schmidhuber, 1997);

(14) $$f_{t}=\sigma \left(b_{t-1}^{T}W_{f}+h_{t-1}^{T}V_{f}+B_{f}\right)$$

(15) $$i_{t}=\sigma \left(b_{t-1}^{T}W_{i}+h_{t-1}^{T}V_{i}+B_{i}\right)$$

(16) $$\tilde{S_{t}}=\text{tanh}\left(b_{t-1}^{T}W_{s}+h_{t-1}^{T}V_{s}+B_{s}\right)$$

(17) $$o_{t}=\sigma \left(b_{t-1}^{T}W_{o}+h_{t-1}^{T}V_{o}+B_{o}\right)$$

(18) $$S_{t}=\sigma \left(f_{t}*S_{t-1}+i_{t}*\tilde{S_{t}}\right)$$

(19) $$h_{t}=o_{t}*tanh\left(S_{t}\right)$$

(20) $${\bar{b}}_{t}=h_{t}^{T}W_{d}+B_{d}$$

where * denotes elementwise multiplication (Hadamard product), + elementwise addition, *W* is the adaptable weights kernel multiplied with *b*_*t*−1_, *V* is the adaptable weights kernel multiplied with *h*_*t–1*_ and *B* is the adaptable bias. *W*_{*f,i,s,o*}_ are all *n*_s_×*d* dimensional real matrices, *V*_{*f,i,s,o*}_> are all *d*×*d* dimensional real matrices and *B*_{*f,i,s,o*}_ are all *d* dimensional real vectors, where *n*_s_ = 5124 is the number of sources. *W*_*d*_ is the adaptable weights kernel matrix of the last densely connected neural network of dimensions *d*×*n*_s_ and *B*_*d*_ is the *n*_s_ dimensional bias vector. $f_{\text{t}},i_{\text{t}},{\tilde{S}}_{\text{t}},o_{t},S_{\text{t}},h_{\text{t}}$ are *d*-dimensional and *b*_t_ is *n*_s_-dimensional. The weight matrices and bias vectors *W, V, B* were trained using supervised learning and is described in section “LSTM Network”.

### Data Analysis

#### Data Acquisition

MEG and MRI data were collected after informed consent from a healthy 27 years old male under a protocol approved by the Massachusetts General Hospital Institutional Review Board. The subject had no medical history of hearing loss.

T1-weighted, high resolution MPRAGE (Magnetization Prepared Rapid Gradient Echo) structural images were acquired on a 1.5 T Siemens whole-body MRI (magnetic resonance) scanner (Siemens Medical Systems) using a 32 channel head coil at MGH.

Auditory steady state response (ASSR) data were recorded from the subject in the MGH Martinos center MEG core in Charlestown, MA, using MEG and EEG. It is the same ASSR data that were used in Samuelsson et al. (2019). The MEG system was an Elekta-Neuromag (Helsinki, Finland) VectorView 306 channel MEG with 102 triplets consisting of one magnetometer and two orthogonal planar gradiometers for a total of 204 planar gradiometers and 102 magnetometers. The EEG was recorded with a 58 channel EasyCap system (EasyCap GmbH, Germany). The experiment was performed in a quiet, magnetically shielded room (Imedco, Switzerland). The recording was bandpass filtered between 0.1 and 1,650 Hz and sampled at 5,000 samples/s. The data were then digitally lowpass filtered at a cutoff frequency of 270 Hz and downsampled to 810 Hz. The ASSRs were elicited by an amplitude modulated (AM) sound which lasted 1 s. The AM sound was followed by an inter-stimulus interval of 500 ms plus jitter that was uniformly distributed between 0 and 750 ms, U(0, 0.75). The sound was thus played to the subject with an inter-trial pause uniformly distributed between 0.5 and 1.25 s duration. The carrier signal was a *f*_0_ =  1kHz sinusoid and was amplitude modulated to a depth of 90% by a superposition of a *f*_1_ =  40Hz and *f*_2_ =  223Hz sinusoid;

(21) $$y\left(t\right)=\left(0.1+0.9\left[\sin\left(2\pi f_{1}t\right)+\sin\left(2\pi f_{2}t\right)\right]/2\right)\sin\left(2\pi f_{0}t\right).$$

#### Data Processing

The structural data were preprocessed using FreeSurfer (Dale et al., 1999; Fischl et al., 1999). After correcting for topological defects, cortical surfaces were tessellated using triangular meshes with ∼130,000 vertices in each hemisphere. To expose the sulci in the visualization of cortical data, we used the inflated surfaces computed by FreeSurfer. 49 bad epochs were dropped using autoreject (Jas et al., 2017), resulting in 1,653 clean, i.e., artifact free, epochs.

#### Forward Model and Inverse Operator

The dense triangulation of the folded cortical surface provided by FreeSurfer was decimated to a grid of 2,562 dipoles per hemisphere, corresponding to a spacing of approximately 6.2 mm between adjacent source locations. A piecewise-homogenous head conductor model with three compartments bounded by the inner skull, outer skull and outer skin was assumed, and the boundary element method (BEM) was used to compute the gain matrix (Hamalainen and Sarvas, 1989). The conductivities were 0.3, 0.006, 0.3 for the brain, skull and scalp, respectively. The watershed algorithm in FreeSurfer was used to generate the tessellations based on the MRI scan of the participant.

The initial current distribution estimate $\hat{q_{t}}$ was obtained using dSPM with loose current dipole orientation constraints set at 0.2, where 0.0 corresponds to fixed and 1.0 to free orientations. The regularized (λ = 0.1) noise covariance matrix used to calculate the inverse operator was calculated over the pre-stimulus period. All forward and inverse calculations were done using MNE-C and MNE-python software (Gramfort et al., 2013a; Esch et al., 2019).

#### Simulation Study

**FIGURE 3:**
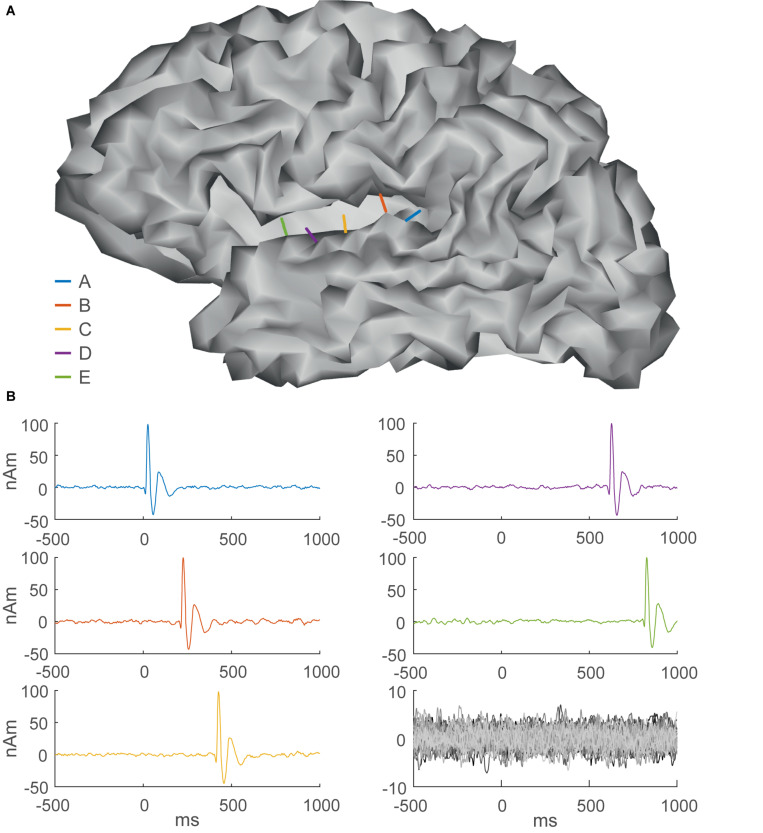
Simulation setup. **(A)** Gray matter surface with five current dipoles in the superior temporal gyrus modeling epileptic foci, color-coded according to their sequential activation pattern as shown in **(B)**. **(B)** Time courses of sequentially activated dipoles (A–E) (color-coded graphs) modeling epileptiform discharges and background activity (lower right). One epoch comprises background activity lasting for 500 ms followed by a propagating epileptiform discharge lasting for 1 s.

A simulation study was conducted to test the performance of CMNE in comparison to dSPM, pure LSTM prediction, a control estimate, mixed-norm estimate (MxNE) and the spatiotemporal Kalman approach estimate as presented in Lamus et al. (2012). In the control estimate, 80 sequential dSPM distributions were averaged and multiplied with the dSPM of the following sample, thus mimicking our contextual estimate but without the LSTM network, which used a lookback of *k* = 80 samples. The gain of using LSTM networks can thus be examined by comparing this control estimate to CMNE. The simulations were designed to mimic the propagation of an epileptiform discharge in the left supratemporal cortex. The source configuration consisted of 5,124 current dipoles placed over the cortex with free orientations. Source space noise was added as stationary Gaussian noise with spectral characteristics taken from EEG readings, as described in Hunold et al. (2016). The activation wave form that modeled the epileptiform discharge was a spike-wave complex lasting 200 ms, starting sequentially in the posterior-anterior direction and making one simulated epileptiform discharge lasting for a total duration of 1,000 ms (Figure 3) and was superimposed on the noise. The spike-wave complex had an amplitude 5 times larger than that of the background activity (10 nAm). The propagating epileptic foci were represented by five current dipoles that were sequentially activated from posterior to anterior locations on the supratemporal cortex with a mutual distance of 9 mm. A total of 250 epileptiform discharges were simulated with a 500 ms interictal period. Figure 3 shows an outline of the dipole activations.

#### Performance Metrics

To evaluate the performance of CMNE and compare it to other related source estimation methods we used the spatial fidelity evaluation metrics presented in Samuelsson et al. (2020); we calculated the peak localization error PE and spatial dispersion SD,

(22) $$\text{PE}={||r_{i}-r_{j}||}_{2},$$

(23) $$j=\underset{i}{\text{argmax}}\left({\left\{\left|{\hat{x}}_{i}\right|\right\}}_{i}\right),$$

(24) $$\text{SD}=\frac{\sum_{k=1}^{N_{s}}d_{jk}\left|{\hat{x}}_{k}\right|}{\sum_{k=1}^{N_{s}}\left|{\hat{x}}_{k}\right|},$$

(25) $$d_{jk}={||r_{j}-r_{k}||}_{2},$$

where *r*_i_ is the location of the active source, *r*_j_ is the location of the peak reconstruction amplitude, *d*_jk_ is the distance between *r*_j_ and source *k* and ${\hat{x}}_{\text{i}}$ is, as before, the estimate at source *i*. To evaluate temporal fidelity, we calculated Pearson’s correlation coefficient *r* in the simulation study between the true source activations (Figure 3) and the time courses of the estimated reconstructions that peaked at the time of the spikes $\hat{x}{\left(t\right)}_{\text{j}}$. We also quantified SNR in source space as

(26) $$\text{SNR}=\frac{\sigma {\left({\left\{\left|{\hat{x}}_{t,i}\right|\right\}}_{i\in A,t\in S}\right)}^{2}}{\sigma {\left({\left\{\left|{\hat{x}}_{t,i}\right|\right\}}_{i\in A,t\notin S}\right)}^{2}},$$

where σ is the standard deviation, *A* is the primary auditory cortex A1 in the ASSR data and the entirety of the auditory cortex in the simulations and *S* is the signal segment defined as the ictal period in the simulations and as the N1 and P2 responses in the ASSR data, i.e., the SNR is defined as the standard deviation of the activation in the auditory cortex during the activation period divided by standard deviation of the activation in the auditory cortex outside of the activation period (Shahin et al., 2007).

### LSTM Network

The LSTM networks were trained on dSPM source estimates. The available epochs were randomly divided up in one training data set (85%) and one validation data set (15%). The training and validation data sets were thus disjoint. The training data were generated using overlapping sliding windows in time over the epochs, each window containing *k* time steps, one for each LSTM cell. The LSTM network predicts the subsequent dSPM activation based on these *k* past time steps and the ground truth is the actual dSPM estimate that it is trying to predict. Prior to the training, the input (past *k* dSPM estimates) and ground truth (current dSPM estimate) were standardized by z-scoring.

We employed the mean-square error (MSE) as the loss function and stochastic gradient descent (Adam algorithm) as the optimization method (Kingma and Ba, 2014). The training was organized in a minibatch setting which split the training process into small batches comprising a small set of gradient evaluations, the LSTM weights being updated using the anti-gradient of the error with respect to the LSTM weights over each minibatch. The LSTM setup, training and evaluation was realized in CNTK/TensorFlow in combination with Keras as the frontend API (Abadi et al., 2015; Seide and Agarwal, 2016; Chollet, 2018).

**FIGURE 4:**
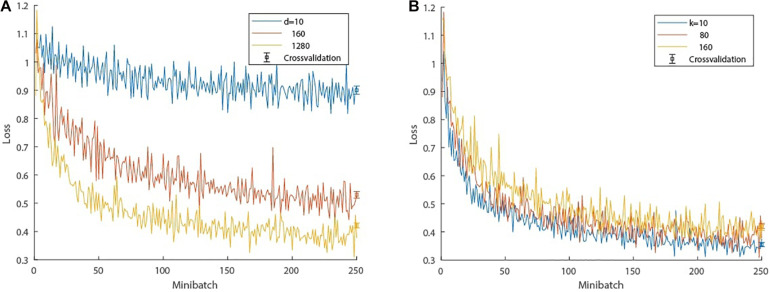
Cross-validation of hyperparameter evaluation. **(A)** Loss progression: influence of the number of hidden units *d* in the LSTM network on the performance with a constant look back of *k=80* samples. **(B)** Loss progression: influence of the look back, i.e., number of past samples *k* used in the LSTM network with a fixed number of hidden units *d=1280*. The bar plots depict the cross-validation testing loss after training. The whiskers denote one standard error and the center refers to the mean loss.

Hyperparameter evaluation (the number of hidden units *d* in the LSTM network and the number of past time steps *k* used as inputs to the LSTM prediction) was done by cross-validation on the ASSR data. In the cross-validation, the training data sets amounted to 85% of all data points and the remaining 15% were used for testing; cross-validation was performed 10 times. The training data were grouped into 30 minibatches. Each minibatch consisted of 30 feature representations, each feature representation being a randomly selected window of 81 consecutive samples, 80 being used as inputs to the LSTM network to predict the 81st sample, which is compared to the ground truth. First, the influence of the LSTM units on the performance was tested by varying *d* with a constant look back of *k=80* samples. The training and testing results are shown in a loss graph in Figure 4A. Second, the optimal number of past time steps *k* was determined with a fixed number of hidden units *d=1280*, which is an appropriate trade-of between prediction accuracy and training time. The results are depicted in Figure 4B, which also offers insight into the genericity of the length of the time window used as input to the LSTM prediction; generally a longer time window results in more accurate predictions but comes at a higher computational cost in the case of the ASSR data. It is conceivable that other brain states elicited by other stimuli could affect these results.

Based on this evaluation, we chose a final LSTM network topology of *d=1280* units and a window size comprising the past *k* = 80 samples. The selection of the number of hidden units was a compromise between training time and prediction accuracy. We anticipate that a larger number of units would further improve the network performance. The choice of *k* was also a trade-off; shorter windows have fewer LSTM cells and thus fewer weights to adjust, leading to faster convergence, while wider time windows are more robust to fluctuations. *k=80* was found to be a suitable trade-off between the two.

## Results

### Simulation Study

In the simulation study, we trained the LSTM network with the topology (*d* = 1280,*k* = 80) using only 100 minibatch iterations. Each minibatch comprised 30 evaluations of 25 windows per evaluation. The windows contained 81 consecutive source estimation samples (80 used as input and 1 as the label), corresponding to a time window of 0.1 s, whose starting points were randomly selected from the 212 raw training epochs.

**FIGURE 5:**
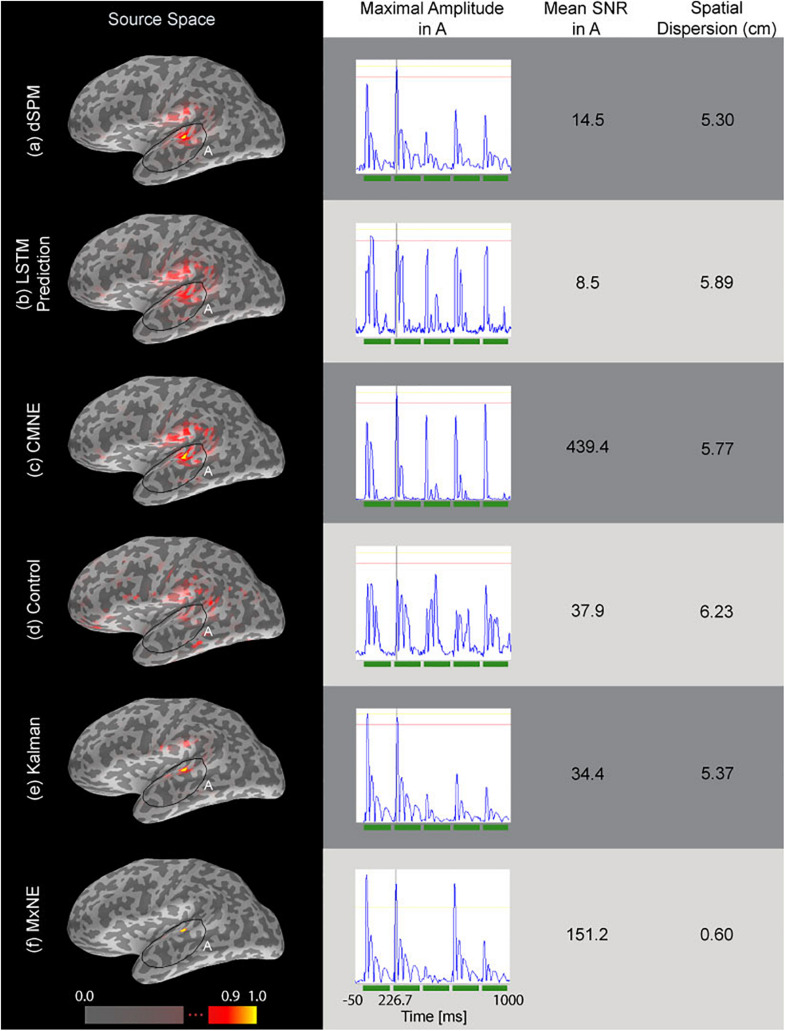
Source estimations based on simulated data (20 averaged epochs) are shown in the source space column. The second column from the left shows the time traces of the estimated dipole with maximal amplitude within the marked area A as depicted in the first column. The third column shows the estimated SNR of the respective source reconstruction method (Eq. 26) and the fourth column shows the spatial dispersion of the estimates (Eq. 24). The tested source estimation methods are divided into rows: **(a)** dSPM estimate ${\hat{q}}_{t}$, **(b)** LSTM network prediction $\bar{b}\left({\hat{q}}_{t}\right)$, **(c)** CMNE estimate $b\left({\hat{q}}_{t}\right)$, **(d)** control estimate, which averages the 80 previous estimates and uses that average to correct the current dSPM estimate based on the average of 20 epochs, **(e)** Kalman estimate, **(f)** MxNE estimate. All estimates were rectified and normalized to their peak value.

Figure 5 shows a comparison between CMNE, dSPM, MxNE, LSTM prediction and a control estimate applied to an average of 20 epochs as well as the Kalman source estimation. CMNE has the highest SNR (Eq. 26) as seen in the third column of Figure 5. However, the LSTM prediction is not able to capture the temporal dynamics of the neural activation patterns adequately; the waveforms get distorted as seen in the second column of Figure 5. The correction step that utilizes the current dSPM estimation compensates for this; the peak of the CMNE estimate is on time but the ripples following the spikes are suppressed. The control estimate has a reduced SNR, significantly lower than the SNR of CMNE, and distorts the waveforms as well as exhibiting a high degree of spatial dispersion. The Kalman source estimate and particularly MxNE result in focal estimates with a low degree of spatial dispersion (column 4), although with lower noise suppression than the CMNE estimate as evident in the lower source space SNR.

**FIGURE 6:**
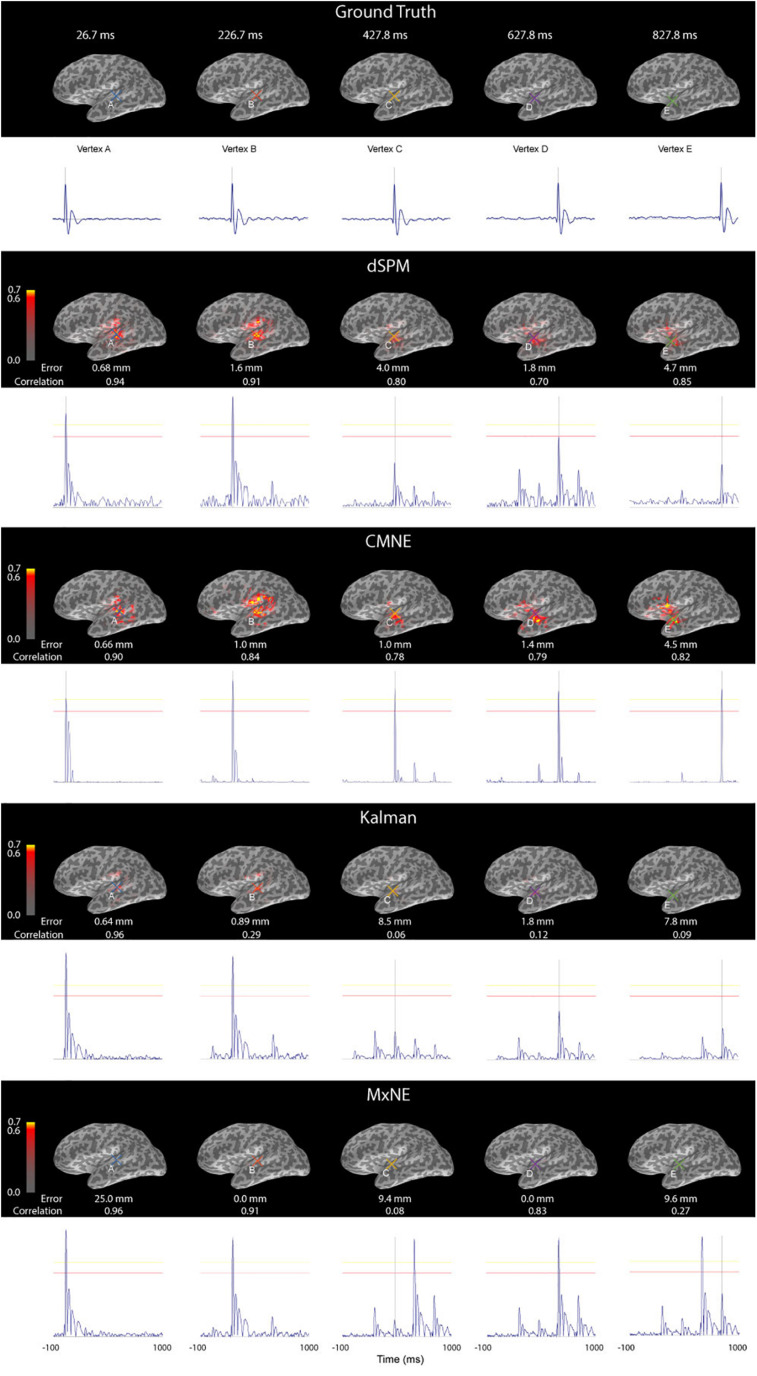
Source activation (first row), dSPM estimate (second row), CMNE estimate (third row), Kalman estimate (fourth row) and MxNE (fifth row) of simulated data displayed over an inflated cortex at the time of peak activations of dipoles (A-E) along with the time traces of the reconstructured dipoles that had the largest amplitudes at the time of peak activations (marked A-E). The results are based on an average of 20 epochs. All estimates were rectified and normalized to their peak value.

Figure 6 shows the source reconstruction by dSPM, CMNE, MxNE, and the Kalman approach at the time of maximal activation along with the ground truth. The localization error PE was lower with CMNE as compared to dSPM for all dipole activations. While MxNE had zero localization error for dipoles B and D, and the Kalman approach resulted in marginally lower localization error than CMNE for dipole activations A and B, CMNE showed a consistent small localization error, being no larger than 4.5 mm for any activation, whereas the other spatiotemporal methods showed a highly variant result, particularly MxNE that resulted in localization errors varying between 0 and 25 mm. We also notice that while dSPM and the Kalman approach do not manage to reconstruct the amplitudes of dipoles C, D, and E, CMNE maintains an adequate amplitude reconstruction for all active vertices. Furthermore, CMNE does not result in spurious activations during interstimulus periods. However, while the activation signals are relatively well reconstructed with dSPM as measured by the relative amplitude of the damped oscillations to the peak value, CMNE concentrates most of the signal energy around the peak activation timepoint, resulting in a less authentic temporal reconstruction, quantified in the Pearson’s correlation coefficient which is higher for dSPM than CMNE for all activations except one. This is, however, a drawback that CMNE shares with the other spatiotemporal methods; while MxNE and the Kalman approach adequately reconstruct the activation in some cases, the reconstruction is completely off in other activations resulting in a very low correlation coefficient.

### Auditory Steady State Response

The same LSTM network topology was used for processing of the ASSR data (*d* = 1280,*k* = 80) and the results were compared with dSPM, MxNE, the Kalman approach and the LSTM prediction alone as well as the control estimate, as was done in the simulation study. The training was performed with 250 minibatch iterations each comprising 30 evaluations containing 20 windows per evaluation. The windows contained 81 subsequent source estimation samples, which were randomly selected from the 1,405 artifact-free epochs used for training. Validation was made based on the remaining 248 epochs that were not used in the training.

**FIGURE 7:**
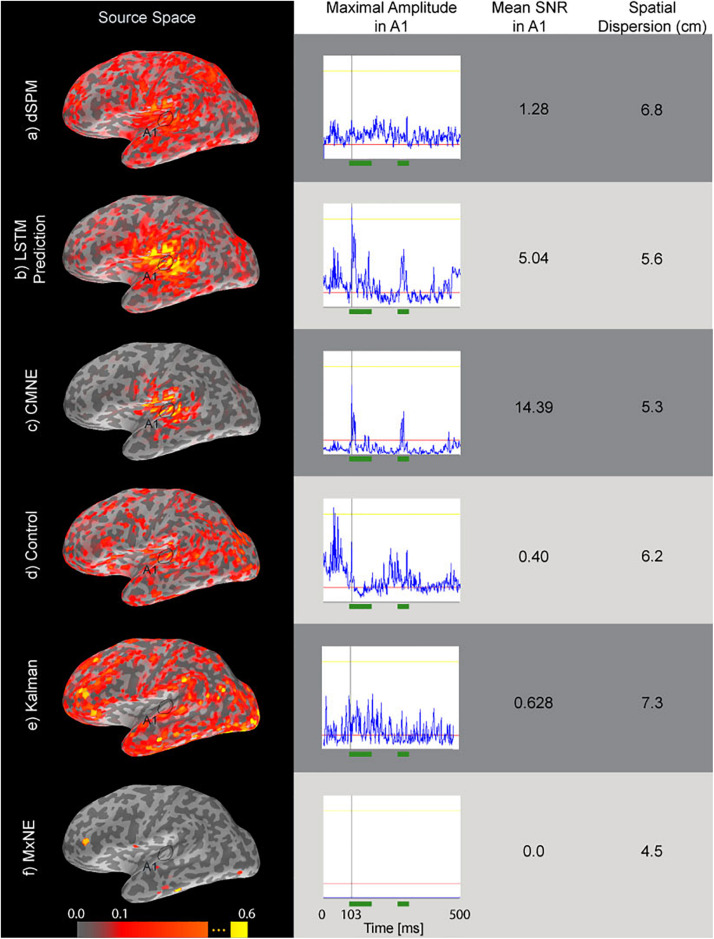
Source estimates (first column) of ASSR data based on averages of 20 epochs, time courses (second column) of the source dipole in A1 with the greatest amplitude over the evoked response, SNR (Eq. 26, third column) and spatial dispersion (Eq. 24, fourth column) of the estimates. The green segments in the time courses mark the N1 and P2 responses that were used in the calculation of SNR (Eq. 26). The rows correspond to dSPM **(a)**, LSTM prediction **(b)**, CMNE **(c)**, control estimate **(d)**, Kalman estimate **(e),** and MxNE **(f)**. All estimates were rectified and normalized to their peak value.

Figure 7 shows the results where the estimates have been found from 20 averaged ASSR epochs. The label A1 marks the primary auditory cortex. The spatial dispersion of CMNE is lower than that with dSPM and results in higher SNR (Eq. 26). The MxNE does not reconstruct any activation in the primary auditory cortex, likely because the input SNR was too low. The Kalman estimate shows higher spatial dispersion than CMNE and does not seem to be able to capture the N1 and P2 responses well. The control estimate resulted in a very inadequate reconstruction, showcasing the added benefit of the LSTM prediction.

## Discussion

The contextual nature of brain activity as evidenced by intracranial electrophysiology and functional connectivity studies (Bullock et al., 1995; Destexhe et al., 1999; Leopold et al., 2003) in addition to the recent advances in employing RNN for processing data with a temporal structure, e.g., natural language processing, inspired the use of LSTM networks in the M/EEG inverse problem for predicting and correcting M/EEG source estimates based on their context. In this study, we developed this technique and applied it to dSPM estimates, naming the implementation CMNE. The approach presented here thus constitutes a novel spatiotemporal inverse method where M/EEG estimates are filtered based on their context without too strong and explicit *a priori* assumptions of their spatiotemporal dynamics.

We tested CMNE on simulated epileptiform and recorded ASSR data, showing that in the cases tested here, the CMNE estimates exhibited a higher SNR in source space (Figures 5, 7), less spatial dispersion (Figure 7) and smaller localization error (Figure 6), thus implying a higher degree of spatial fidelity, than the unfiltered dSPM estimates. The other spatiotemporal methods tested in this study was the Kalman approach proposed by Lamus et al. (2012) and MxNE. While MxNE and the Kalman approach showed a high degree of spatial fidelity in some cases, manifested in very low localization error for some activations and low spatial dispersion in the simulation study, the simulation also showed that their performance was highly variant, e.g., the reconstruction error with MxNE varied between 0 and 25 mm. In comparison to the other spatiotemoral methods, CMNE thus showed a more consistent performance. That was also observed in the temporal fidelity comparison which showed that CMNE had a Pearson’s correlation coefficient consistently above 0.78 with the true activation signal while the Kalman and mixed norm approaches resulted in a correlation varying between 0.06 and 0.96. Furthermore, the assessed source space SNR was significantly higher with CMNE than in any of the other approaches tested here because of the temporal sparsity. This ability of CMNE to extract the signal from noisy data could be the reason for the superior performance on the ASSR data, where the Kalman and MxNE approaches performed substantially worse than CMNE in spite of the steady local activity in the auditory cortex that occurs during ASSR where one might think the Kalman and MxNE approaches would do better.

However, the reconstructed time courses of the sources as predicted by the LSTM network were not always able to follow steep ascends, which relates to a lowpass characteristic (Figures 5, 6). Correcting the prediction with the dSPM estimate based on the sensor data resulted in a higher degree of temporal fidelity than the LSTM prediction. Even after the correction, however, the damped oscillations following the spikes in the simulation study were not faithfully reconstructed. The CMNE filtering thus resulted in a distortion of the waveform following the spike, which does not take place to the same extent with dSPM. This highlights a drawback of CMNE; although we can achieve enhanced spatial fidelity, the fact that we have a spatial filter that changes over time can introduce a temporal phase shift which could distort the waveform reconstruction. This is, however, a characteristic that CMNE shares with other spatiotemporal methods and they must therefore be used with caution when doing temporal analysis, e.g., examining frequency bands or performing phase synchrony analysis. How to introduce a zero-phase shift spatial filter based upon spatiotemporal information without explicit *a priori* assumptions, such as the technique presented here, should be the topic of a future study.

In the simulation study, the activation pattern was in the form of dipoles at different locations activated subsequently. This activation pattern should be predictable and the CMNE estimation should therefore give better results over time as progressively more information on past activity is gathered since the internal cell state of each LSTM cell is passed forward to the next cell in the chain. This is indeed the case, as it can be seen from Figure 6 that while the dSPM estimate exhibited an increasingly higher degree of spatial dispersion, the CMNE estimate maintained an adequate amplitude reconstruction. It should also be noted that we here used the same source mesh and field computation method in the forward modeling as in the inverse modeling, which resulted in low localization errors for some of the inverse methods.

There is no definite rule how to select the number of neurons in each neural network layer *d* and the number of LSTM cells *k*; cross-validation is normally employed to select these hyperparameters. Due to limitations in computational resources and the difficulties in presenting results for many different hyperparameter values, we resorted here to the conventional approach of evaluating hyperparameters by cross-validation first and then employing these hyperparameters throughout the study. Further evaluation of how the choice of network topology could influence the result should be explored in future studies. How well these hyperparameters will generalize to unseen data, such as a new subject or measurement system or evoked response, will depend upon the spatiotemporal distribution of the new data.

The distributed source estimates found with dSPM that we use as inputs to the network makes the input data somewhat more invariant across subjects and measurement systems than if we had used sensor space data, which would likely not have generalized well across measurement systems. However, different subjects, evoked responses and measurement systems might still require new training of a subset of the weights. It is also possible that this spatial and temporal variability could be addressed by using a larger *k* and *d* to account for temporal and spatial variability, respectively. Larger LSTM networks with a high dropout rate (>0.7) might thus improve the generalization of CMNE by increasing the complexity of the model and letting a high dropout rate mitigate overfitting. This would, however, also increase the computational complexity of the training which is already quite high (∼8–12 h on a conventional workstation with dual XEON E5-2687W, 64 GB RAM, and NVIDIA Quadro 4000). Efficient transfer learning schemes with automatic hyperparameter search could potentially be employed as an alternative (Yogatama and Mann, 2014). It would also be of great interest to see how the weights in the LSTM network differs in different evoked responses. It is conceivable that the internal cell states that are modulated by the weights carry information about the active brain state. How these are modulated in different tasks that activate different functional networks is an interesting aspect of this technique that needs further investigation.

Another aspect that warrants further investigation is combining the CMNE approach used here with other spatiotemporal source estimates. For instance, this approach could be combined with Kalman filtering, e.g., where a Kalman or extended Kalman filter could be used to update the weights in the LSTM network, instead of backpropagation.

Finally, there is a wide variety of neural networks and it is hard to predict, without empirical testing, which implementation and combination of networks that would yield the optimal performance given a measure of goodness. It is conceivable that, for instance, bi-directional LSTM networks using both past and future estimates would capture the temporal dynamics better than the unidirectional LSTM network implemented in this study, or that convolutional neural networks (CNN), commonly used in computer vision, could be used to increase the spatial fidelity even further. A fundamental issue with implementing machine learning methods in M/EEG source estimation is the lack of ground truth data. It also remains unclear to what degree the networks can be trained on a variety of subjects and tasks or if they should be trained separately as was done here. Addressing these questions will be critical for the future of machine learning in M/EEG source estimation.

## Conclusion

A novel technique was introduced where a spatiotemporal LSTM network is used to predict the source estimate following a sequence of past estimates. The prediction is then used as a spatial filter to correct the estimate, which is context dependent since it is a function of past estimates. Because this technique can be used in conjunction with any source estimation method that has a temporal sequence and does not rest on strong and explicit *a priori* modeling assumptions, any source estimation method can be turned into a spatiotemporal method using the technique presented here. We tested an implementation of this technique on dSPM estimates, naming it CMNE, and benchmarked it on simulations of ictal events and recorded M/EEG data from an ASSR experiment, showing that it can result in a higher degree of spatial fidelity as compared to the unfiltered, non-contextual estimates. We conclude that the results shown here indicate promise for the emerging field of the application of machine learning in M/EEG source estimation and warrant more studies on different network configurations and training procedures.

## Data Availability Statement

The raw data supporting the conclusions of this article will be made available by the authors, without undue reservation, to any qualified researcher.

## Ethics Statement

The studies involving human participants were reviewed and approved by Massachusetts General Hospital Institutional Review Board. The patients/participants provided their written informed consent to participate in this study.

## Author Contributions

CD conceptualized and designed the study and performed the data visualization. JS and SK collected the ASSR data. AH performed the epileptiform simulations. CD and JS performed the analysis. CD, JS, and MH wrote the first draft of the manuscript. MH contributed funding. All authors contributed to manuscript revision, read, and approved the submitted version.

## Conflict of Interest

The authors declare that the research was conducted in the absence of any commercial or financial relationships that could be construed as a potential conflict of interest.

## References

[B1] Abadi M. Agarwal A. Barham P. Brevdo E. Chen Z. Citro C. (2015). *TensorFlow**: Large-Scale Machine Learning on Heterogeneous Systems.* Available online at: https://arxiv.org/abs/1603.04467 (accessed December 1, 2017).

[B2] Ali H. Karim F. Qureshi J. J. Abuassba A. O. Bulbul M. F. (2019). Seizure prediction using bidirectional LSTM. in *Proceedings of the International Conference Cyberspace Data and Intelligence, and Cyber-Living, Syndrome, and Health.* Berlin: Springer, 349–356. 10.1007/978-981-15-1922-2_25

[B3] Baillet S. Garnero L. (1997). A bayesian approach to introducing anatomo-functional priors in the EEG/MEG inverse problem. *IEEE Trans. Biomed. Eng.* 44 374–385. 10.1109/10.568913 9125822

[B4] Bolstad A. Van Veen B. Nowak R. (2009). Space–time event sparse penalization for magneto-/electroencephalography. *Neuroimage* 46 1066–1081. 10.1016/j.neuroimage.2009.01.056 19457366 PMC2850823

[B5] Bullock T. McClune M. Achimowicz J. Iragui-Madoz V. Duckrow R. Spencer S. (1995). Temporal fluctuations in coherence of brain waves. *Proc. Natl. Acad. Sci. U.S.A.* 92 11568–11572. 10.1073/pnas.92.25.11568 8524805 PMC40443

[B6] Calvetti D. Somersalo E. Strang A. (2019). Hierachical bayesian models and sparsity: l 2-magic. *Inverse Probl.* 35:035003.

[B7] Chollet F. (2018). *Keras: The Python Deep Learning Library.* Astrophysics Source Code Library. Available online at: https://ui.adsabs.harvard.edu/abs/2018ascl.soft06022C/abstract (accessed June, 2018).

[B8] Chorowski J. K. Bahdanau D. Serdyuk D. Cho K. Bengio Y. (2015). “Attention-based models for speech recognition,” in *Advances in Neural Information Processing Systems*, eds Jordan M. I. LeCun Y. Solla S. A. (Cambridge, MA: MIT Press).

[B9] Costa F. Batatia H. Oberlin T. d’Giano C. Tourneret J.-Y. (2017). Bayesian EEG source localization using a structured sparsity prior. *Neuroimage* 144 142–152. 10.1016/j.neuroimage.2016.08.064 27639353

[B10] Dale A. M. Fischl B. Sereno M. I. (1999). Cortical surface-based analysis. i. segmentation and surface reconstruction. *Neuroimage* 9 179–194. 9931268 10.1006/nimg.1998.0395

[B11] Dale A. M. Liu A. K. Fischl B. R. Buckner R. L. Belliveau J. W. Lewine J. D. (2000). Dynamic statistical parametric mapping: combining fMRI and MEG for high-resolution imaging of cortical activity. *Neuron* 26 55–67. 10798392 10.1016/s0896-6273(00)81138-1

[B12] Destexhe A. Contreras D. Steriade M. (1999). Spatiotemporal analysis of local field potentials and unit discharges in cat cerebral cortex during natural wake and sleep states. *J. Neurosci.* 19 4595–4608. 10.1523/jneurosci.19-11-04595.1999 10341257 PMC6782626

[B13] Dinh C. (2015). *Brain monitoring: Real-time Localization of Neuronal Activity. 1st edn.* Aachen: Shaker. 10.2370/9783844038378

[B14] Dinh C. Esch L. Rühle J. Bollmann S. Güllmar D. Baumgarten D. (2018). Real-time clustered multiple signal classification (RTC-MUSIC). *Brain Topogr.* 31 125–128. 10.1007/s10548-017-0586-7 28879632 PMC5773364

[B15] Dinh C. Samuelsson J. G. Hunold A. Hämäläinen M. S. Khan S. (2019). Contextual minimum-norm estimates (CMNE): a deep learning method for source estimation in neuronal networks. *arXiv [preprint]* arXiv1909.02636.

[B16] Dinh C. Strohmeier D. Luessi M. Gullmar D. Baumgarten D. Haueisen J. (2015). Real-time MEG source localization using regional clustering. *Brain Topogr.* 28 771–784. 10.1007/s10548-015-0431-9 25782980 PMC4575234

[B17] Esch L. Dinh C. Larson E. Engemann D. Jas M. Khan S. (2019). “MNE: software for acquiring, processing, and visualizing MEG/EEG data,” in *Magnetoencephalography: From Signals to Dynamic Cortical Networks*, eds Supek S. Aine C. J. (Berlin: Springer), 355–371. 10.1007/978-3-030-00087-5_59

[B18] Fischl B. Sereno M. I. Dale A. M. (1999). Cortical surface-based analysis. II: inflation, flattening, and a surface-based coordinate system. *Neuroimage* 9 195–207. 10.1006/nimg.1998.0396 9931269

[B19] Friston K. Harrison L. Daunizeau J. Kiebel S. Phillips C. Trujillo-Barreto N. (2008). Multiple sparse priors for the M/EEG inverse problem. *Neuroimage* 39 1104–1120. 10.1016/j.neuroimage.2007.09.048 17997111

[B20] Galka A. Yamashita O. Ozaki T. Biscay R. Valdés-Sosa P. (2004). A solution to the dynamical inverse problem of EEG generation using spatiotemporal kalman filtering. *Neuroimage* 23 435–453. 10.1016/j.neuroimage.2004.02.022 15488394

[B21] Gorodnitsky I. F. George J. S. Rao B. D. (1995). Neuromagnetic source imaging with FOCUSS: a recursive weighted minimum norm algorithm. *Electroencephalogr. Clin. Neurophysiol.* 95 231–251. 10.1016/0013-4694(95)00107-a 8529554

[B22] Gramfort A. Kowalski M. Hamalainen M. (2012). Mixed-norm estimates for the M/EEG inverse problem using accelerated gradient methods. *Phys. Med. Biol.* 57 1937–1961. 10.1088/0031-9155/57/7/1937 22421459 PMC3566429

[B23] Gramfort A. Luessi M. Larson E. Engemann D. A. Strohmeier D. Brodbeck C. (2013a). MEG and EEG data analysis with MNE-Python. *Front. Neurosci.* 7:267. 10.3389/fnins.2013.00267 PMC3872725 24431986

[B24] Gramfort A. Strohmeier D. Haueisen J. Hamalainen M. S. Kowalski M. (2013b). Time-frequency mixed-norm estimates: sparse M/EEG imaging with non-stationary source activations. *Neuroimage* 70 410–422. 10.1016/j.neuroimage.2012.12.051 23291276 PMC3615257

[B25] Graves A. Mohamed A.-R. Hinton G. (2013). “Speech recognition with deep recurrent neural networks,” in *Proceedings of the 2013 IEEE International Conference on Acoustics, Speech and Signal Processing*, (Pisacataway, NJ: IEEE), 6645–6649.

[B26] Greensite F. (2003). The temporal prior in bioelectromagnetic source imaging problems. *IEEE Trans. Biomed. Eng.* 50 1152–1159. 10.1109/tbme.2003.817632 14560768

[B27] Gross J. Kujala J. Hämäläinen M. Timmermann L. Schnitzler A. Salmelin R. (2001). Dynamic imaging of coherent sources: studying neural interactions in the human brain. *Proc. Natl. Acad. Sci. U.S.A.* 98 694–699. 10.1073/pnas.98.2.694 11209067 PMC14650

[B28] Hamalainen M. S. Ilmoniemi R. J. (1994). Interpreting magnetic-fields of the brain - minimum norm estimates. *Med. Biol. Eng. Comput.* 32 35–42. 10.1007/bf02512476 8182960

[B29] Hamalainen M. S. Sarvas J. (1989). Realistic conductivity geometry model of the human head for interpretation of neuromagnetic data. *IEEE Trans. Biomed. Eng.* 36 165–171. 10.1109/10.16463 2917762

[B30] Hämäläinen M. Hari R. Ilmoniemi R. J. Knuutila J. Lounasmaa O. V. (1993). Magnetoencephalography - theory, instrumentation, and applications to noninvasive studies of the working human brain. *Rev. Mod. Phys.* 65:413. 10.1103/revmodphys.65.413

[B31] Helmholtz H. V. (1853). Ueber einige gesetze der vertheilung elektrischer ströme in körperlichen leitern, mit anwendung auf die thierisch-elektrischen versuche (schluss.). *Annalen der Physik* 165 353–377. 10.1002/andp.18531650702

[B32] Hochreiter S. Schmidhuber J. (1997). Long short-term memory. *Neural Comput.* 9 1735–1780. 9377276 10.1162/neco.1997.9.8.1735

[B33] Hofmann S. M. Klotzsche F. Mariola A. Nikulin V. V. Villringer A. Gaebler M. (2018). “Decoding subjective emotional arousal during a naturalistic VR experience from EEG using LSTMs,” in *Proceedings of the 2018 IEEE International Conference on Artificial Intelligence (AIVR)*, (Piscataway, NJ: IEEE), 128–131.

[B34] Hunold A. Funke M. E. Eichardt R. Stenroos M. Haueisen J. (2016). EEG and MEG: sensitivity to epileptic spike activity as function of source orientation and depth. *Physiol. Meas.* 37 1146–1162. 10.1088/0967-3334/37/7/1146 27328313

[B35] Izhikevich E. M. Edelman G. M. (2008). Large-scale model of mammalian thalamocortical systems. *Proc. Natl. Acad. Sci. U.S.A.* 105 3593–3598. 10.1073/pnas.0712231105 18292226 PMC2265160

[B36] Jas M. Engemann D. A. Bekhti Y. Raimondo F. Gramfort A. (2017). Autoreject: automated artifact rejection for MEG and EEG data. *Neuroimage* 159 417–429. 10.1016/j.neuroimage.2017.06.030 28645840 PMC7243972

[B37] Jirsa V. K. Jantzen K. J. Fuchs A. Kelso J. S. (2002). Spatiotemporal forward solution of the EEG and MEG using network modeling. *IEEE Trans. Med. Imaging* 21 493–504. 10.1109/tmi.2002.1009385 12071620

[B38] Khan S. Michmizos K. Tommerdahl M. Ganesan S. Kitzbichler M. Zetino M. (2015). Somatosensory cortex functional connectivity abnormalities in autism show opposite trends, depending on direction and spatial scale. *Brain* 138(Pt 5), 1394–1409. 10.1093/brain/awv043 25765326 PMC5013931

[B39] Kingma D. P. Ba J. (2014). Adam: a method for stochastic optimization. *Comput. Sci. Mach. Learn.* 1–15. Available online at: http://arxiv.org/abs/1412.6980 (accessed May 5, 2018).

[B40] Kozhemiako N. Nunes A. Samal A. Rana K. Calabro F. Hämäläinen M. (2020). Neural activity underlying the detection of an object movement by an observer during forward self-motion: dynamic decoding and temporal evolution of directional cortical connectivity. *Prog. Neurobiol.* 195:101824. 10.1016/j.pneurobio.2020.101824 32446882 PMC7680311

[B41] Lamus C. Hamalainen M. S. Temereanca S. Brown E. N. Purdon P. L. (2012). A spatiotemporal dynamic distributed solution to the MEG inverse problem. *Neuroimage* 63 894–909. 10.1016/j.neuroimage.2011.11.020 22155043 PMC3432302

[B42] LeCun Y. Bengio Y. Hinton G. (2015). Deep learning. *Nature* 521 436–444. 26017442 10.1038/nature14539

[B43] Leopold D. A. Murayama Y. Logothetis N. K. (2003). Very slow activity fluctuations in monkey visual cortex: implications for functional brain imaging. *Cereb. Cortex* 13 422–433. 10.1093/cercor/13.4.422 12631571

[B44] Limpiti T. Van Veen B. D. Attias H. T. Nagarajan S. S. (2008). A spatiotemporal framework for estimating trial-to-trial amplitude variation in event-related MEG/EEG. *IEEE Trans. Biomed. Eng.* 56 633–645. 10.1109/tbme.2008.2008423 19272883 PMC2756105

[B45] Long C. J. Purdon P. L. Temereanca S. Desai N. U. Hamalainen M. S. Brown E. N. (2011). State-space solutions to the dynamic magnetoencephalography inverse problem using high performance computing. *Ann. Appl. Stat.* 5 1207–1228. 10.1214/11-aoas483 22081780 PMC3212953

[B46] Lucka F. Pursiainen S. Burger M. Wolters C. H. (2012). Hierarchical Bayesian inference for the EEG inverse problem using realistic FE head models: depth localization and source separation for focal primary currents. *Neuroimage* 61 1364–1382. 10.1016/j.neuroimage.2012.04.017 22537599

[B47] Mattout J. Phillips C. Penny W. D. Rugg M. D. Friston K. J. (2006). MEG source localization under multiple constraints: an extended Bayesian framework. *Neuroimage* 30 753–767. 10.1016/j.neuroimage.2005.10.037 16368248

[B48] Nummenmaa A. Auranen T. Hamalainen M. S. Jaaskelainen I. P. Lampinen J. Sams M. (2007). Hierarchical Bayesian estimates of distributed MEG sources: theoretical aspects and comparison of variational and MCMC methods. *Neuroimage* 35 669–685. 10.1016/j.neuroimage.2006.05.001 17300961

[B49] Olah C. (2015). *Understanding Lstm Networks.* Available online at: http://colah.github.io/posts/2015-08-Understanding-LSTMs/ (accessed March 17, 2018).

[B50] Ou W. Hamalainen M. S. Golland P. (2009). A distributed spatio-temporal EEG/MEG inverse solver. *Neuroimage* 44 932–946. 10.1016/j.neuroimage.2008.05.063 18603008 PMC2730457

[B51] Pascual-Marqui R. D. (2002). Standardized low-resolution brain electromagnetic tomography (sLORETA): technical details. *Methods Find. Exp. Clin. Pharmacol.* 24 5–12. 12575463

[B52] Pascual-Marqui R. D. Michel C. M. Lehmann D. (1994). Low resolution electromagnetic tomography: a new method for localizing electrical activity in the brain. *Int. J. Psychophysiol.* 18 49–65. 10.1016/0167-8760(84)90014-x 7876038

[B53] Phillips C. Mattout J. Rugg M. D. Maquet P. Friston K. J. (2005). An empirical Bayesian solution to the source reconstruction problem in EEG. *Neuroimage* 24 997–1011. 10.1016/j.neuroimage.2004.10.030 15670677

[B54] Pirondini E. Babadi B. Obregon-Henao G. Lamus C. Malik W. Q. Hamalainen M. S. (2018). Computationally efficient algorithms for sparse, dynamic solutions to the Eeg source localization problem. *IEEE Trans. Biomed. Eng.* 65 1359–1372. 10.1109/tbme.2017.2739824 28920892

[B55] Samuelsson J. G. Khan S. Sundaram P. Peled N. Hämäläinen M. S. (2019). Cortical Signal suppression (CSS) for detection of subcortical activity using MEG and EEG. *Brain Topogr.* 32 215–228. 10.1007/s10548-018-00694-5 30604048 PMC6374174

[B56] Samuelsson J. G. Peled N. Mamashli F. Ahveninen J. Hämäläinen M. S. (2020). Spatial fidelity of MEG/EEG source estimates: a general evaluation approach. *Neuroimage* 224:117430. 10.1016/j.neuroimage.2020.117430 33038537 PMC7793168

[B57] Sato M.-A. Yoshioka T. Kajihara S. Toyama K. Goda N. Doya K. (2004). Hierarchical Bayesian estimation for MEG inverse problem. *Neuroimage* 23 806–826. 10.1016/j.neuroimage.2004.06.037 15528082

[B58] Schmidhuber J. (2015). Deep learning in neural networks: an overview. *Neural Netw.* 61 85–117. 10.1016/j.neunet.2014.09.003 25462637

[B59] Seide F. Agarwal A. (2016). “CNTK: Microsoft’s open-source deep-learning toolkit,” in *Proceedings of the 22nd ACM SIGKDD International Conference on Knowledge Discovery and Data Mining*, (New York, NY: Association for Computing Machinery).

[B60] Shahin A. J. Roberts L. E. Miller L. M. McDonald K. L. Alain C. (2007). Sensitivity of EEG and MEG to the N1 and P2 auditory evoked responses modulated by spectral complexity of sounds. *Brain Topogr.* 20 55–61. 10.1007/s10548-007-0031-4 17899352 PMC4373076

[B61] Somersalo E. Voutilainen A. Kaipio J. (2003). Non-stationary magnetoencephalography by Bayesian filtering of dipole models. *Inverse Probl.* 19:1047. 10.1088/0266-5611/19/5/304

[B62] Sorrentino A. Parkkonen L. Pascarella A. Campi C. Piana M. (2009). Dynamical MEG source modeling with multi-target Bayesian filtering. *Hum. Brain Mapp.* 30 1911–1921. 10.1002/hbm.20786 19378276 PMC6870724

[B63] Strohmeier D. Haueisen J. Gramfort A. (2014). “Improved MEG/EEG source localization with reweighted mixed-norms,” in *Proceedings of the 2014 International Workshop on Pattern Recognition in Neuroimaging*, (Piscataway, NJ: IEEE), 1–4.

[B64] Trujillo-Barreto N. J. Aubert-Vázquez E. Penny W. D. (2008). Bayesian M/EEG source reconstruction with spatio-temporal priors. *Neuroimage* 39 318–335. 10.1016/j.neuroimage.2007.07.062 17904869

[B65] Vivaldi V. Sorrentino A. (2016). Bayesian smoothing of dipoles in magneto-/electroencephalography. *Inverse Probl.* 32:045007. 10.1088/0266-5611/32/4/045007

[B66] Wright J. Rennie C. Lees G. Robinson P. Bourke P. Chapman C. (2003). Simulated electrocortical activity at microscopic, mesoscopic, and global scales. *Neuropsychopharmacology* 28 S80–S93. 12827148 10.1038/sj.npp.1300138

[B67] Yogatama D. Mann G. (2014). “Efficient transfer learning method for automatic hyperparameter tuning,” *Proceedings of the Seventeenth International Conference on Artificial Intelligence and Statistics*, 33 1077–1085. Proceedings of the Seventeenth International Conference on Artificial Intelligence and Statistics, PMLR 33:1077-1085, 2014.

[B68] Yu B.-W. Jeong J.-H. Lee D.-H. Lee S.-W. (2019). “Detection of pilot’s drowsiness based on multimodal convolutional bidirectional LSTM network,” in *Proceedings of the Asian Conference on Pattern Recognition*, (Berlin: Springer).

[B69] Zumer J. M. Attias H. T. Sekihara K. Nagarajan S. S. (2008). Probabilistic algorithms for MEG/EEG source reconstruction using temporal basis functions learned from data. *Neuroimage* 41 924–940. 10.1016/j.neuroimage.2008.02.006 18455439 PMC4361188

